# TRH site-specific methylation in oral and oropharyngeal squamous cell carcinoma

**DOI:** 10.1186/s12885-018-4706-x

**Published:** 2018-08-06

**Authors:** C. Puttipanyalears, A. Arayataweegool, K. Chalertpet, P. Rattanachayoto, P. Mahattanasakul, N. Tangjaturonsasme, V. Kerekhanjanarong, A. Mutirangura, N. Kitkumthorn

**Affiliations:** 1 Department of Anatomy, Faculty of Medicine, Center of Excellence in Molecular Genetics of Cancer and Human Diseases, Chulalongkorn University, Bangkok, 10330 Thailand; 2 Division of Medical Oncology, Department of Medicine, Chulalongkorn University and The King Chulalongkorn Memorial Hospital, Bangkok, 10330 Thailand; 3 Department of Otolaryngology, Head and Neck Surgery, Faculty of Medicine, Chulalongkorn University, Pathumwa, Bangkok, 10330 Thailand; 4 Department of Otolaryngology, Head and Neck Surgery, King Chulalongkorn Memorial Hospital, Thai Red Cross Society, Pathumwan, Bangkok, 10330 Thailand; 5 Department of Oral Biology, Faculty of Dentistry, Mahidol University, 6 Yothi Road, Ratchathewi, Bangkok, 10400 Thailand

**Keywords:** Oral cancer, DNA methylation, Bioinformatics, Pyrosequencing

## Abstract

**Background:**

The incidence of oral squamous cell carcinoma (OSCC) continues to increase each year. Clinical examination and biopsy usually detect OSCC at an advanced stage that is difficult to treat, leading to poor prognosis. DNA methylation pattern is tissue specific and has emerged as a biomarker for the detection of cancers of tissue origin. Herein, we aimed to discover a novel site-specific methylation marker for OSCC.

**Methods:**

We selected OSCC datasets analyzed using the IlluminaHumanMethylation27 BeadChip from the Gene Expression Omnibus repository of the National Center for Biotechnology Information using a bioinformatics approach. From 27,578 CG dinucleotide (CpG) sites, the CpG site with the highest difference in methylation level between healthy and cancerous cells was selected for further validation. A total of 18 mucosal tissue samples were collected from nine healthy controls and nine from OSCC subjects and subjected to microdissection for cell purification, followed by DNA extraction, bisulfite conversion, and pyrosequencing. Additionally, epithelial cells were collected from 2 cohorts including oral rinse from healthy controls, oral rinse and oral swab from OSCC subjects and oral rinse from oropharyngeal squamous cell carcinoma (SCC) were examined for their methylation status using real-time polymerase chain reaction (PCR).

**Results:**

Among the 27,578 differentially methylated CpG sites, cg01009664 of the thyrotropin-releasing hormone (TRH) gene showed the greatest difference in methylation level between healthy and cancerous cells. Validation of the TRH gene using pyrosequencing revealed a methylation percentage of 7% ± 3.43% in healthy cells in contrast to 63% ± 19.81% in cancerous cells. Screening of epithelial cells using real-time PCR showed that the DNA methylation level was significantly higher in oral swab and rinse samples collected from OSCC and oropharyngeal SCC subjects than those from healthy controls (*p* < 0.001). In addition, when using a cutoff at 3.31 ng/μL, the TRH methylation biomarker was able to distinguish OSCC and oropharyngeal SCC subjects from healthy controls with high level of area under the curve, sensitivity and specificity.

**Conclusion:**

We demonstrated cg01009664 of TRH as a potential biomarker for OSCC and oropharyngeal SCC screening using oral rinse and swab techniques.

**Electronic supplementary material:**

The online version of this article (10.1186/s12885-018-4706-x) contains supplementary material, which is available to authorized users.

## Background

Oral squamous cell carcinoma (OSCC) is a major health issue; its incidence rate has continually increased in recent years and is ranked the sixth highest by the National Cancer Institute of Thailand [1]. Clinical signs of OSCC vary between individuals. OSCC is usually detected at an advanced stage via conventional gold standard methods, including clinical examination and biopsy [2]. Biopsy is a relatively painful and invasive procedure that affects patients both physically and psychologically. To reduce the adverse effects of biopsy and to promote optimal treatment plan for OSCC, DNA methylation patterns of genes used as biomarkers for the early diagnosis of OSCC and, consequently, a better prognosis of patients’ health [3].

In recent years, some studies have investigated promoter methylations of tumor suppressor genes to develop putative screening markers in oral rinse and saliva of OSCC patients. However, either sensitivity or specificity level of these markers was not completely satisfied [4–6]. This study designed to focus on CG dinucleotide (CpG) site-specific methylation.

In the beginning, we identified DNA methylation biomarkers for OSCC using a bioinformatics approach. From the methylation microarray data previously deposited in the National Center for Biotechnology Information (NCBI) (www.ncbi.nlm.nih.gov), we selected data representing the methylation profiles in OSCC and head and neck squamous cell carcinoma (HNSCC) tissues and identified CpG sites showing differential methylation patterns between healthy and cancerous tissues. The selected marker was subsequently validated in clinical samples of oral rinse and swab as a screening test for early diagnosis.

## Methods

### Ethics statement

The research methodology employed in this study was approved by The Institutional Review Board of the Faculty of Medicine, Chulalongkorn University, Bangkok, Thailand (IRB No. 426/58 and 135/59). All study subjects provided written informed consent.

### Bioinformatics

**Fig. 1:**
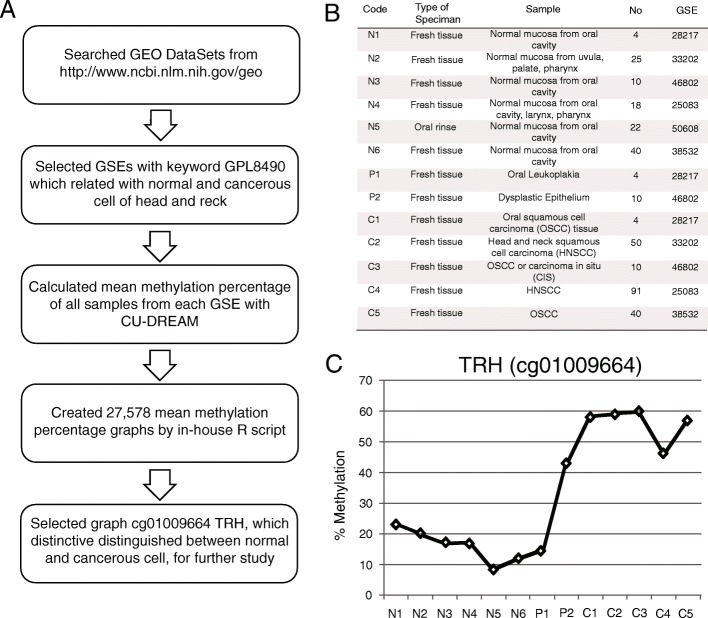
Illustrations of bioinformatics part. **a** Flowchart of bioinformatics approach. **b** Data of 13 sample groups including type of specimen, detail of specimen, number of specimen, and GSE correspondence. Code for sample groups were N; normal, P; oral potential malignant lesion and C; carcinoma. **c** Methylation levels at cg01009664 of TRH presented the increasing level in carcinoma samples (C1-C5)

The bioinformatics pipeline used in this study is shown in Fig. 1a. Methylation microarray data were retrieved from the Gene Expression Omnibus (GEO) repository of NCBI (http://www.ncbi.nlm.nih.gov) [7]. These data contained the Series as GSExxxx, Platform as GPLxxxx, and Sample accession numbers as GSMxxxx. The methylation profiling array Platform, GPL8490 (Illumina® HumanMethylation27 BeadChip Kit, Illumina Inc., San Diego, CA, USA), was used to search for GSE data related with the following keywords: head and neck cancer, HNSCC, OSCC, and normal oral epithelial cell. The GPL8490 methylation profiling array Platform was selected because it has been widely used and provides more data than other microarrays [8, 9]. Samples from each GSE were analyzed according to the inclusion criteria (normal epithelium, precancerous epithelium, and cancerous epithelium of head and neck), and exclusion criteria (cell line, stem cell, non-human tissue, non-head and non-neck tissue sample, inflammation, congenital disease, and blood cells). Based on these criteria, data were narrowed down to seven GSEs (by the end of July 31, 2015) as shown in Fig. 1b [6, 10–13]. The Connection Up- and Down-Regulation Expression Analysis of Microarrays program [14] was used to calculate the average methylation percentage of differentially methylated CpG sites of each sample from the seven GSEs; these data were recorded in Microsoft Office Excel (Additional file 1). The average methylation percentages of normal, precancerous, and cancerous cells were plotted for each CpG site using R.

### Study subjects

To validate the data obtained using bioinformatics, a total of 18 subjects, including 9 healthy controls (four males and five females) and 9 OSCC subjects (five males and four females) were recruited. The samples were supported by the Department of Pathology, Faculty of Medicine, Chulalongkorn University, Bangkok, Thailand. Tissues of gum were collected from healthy controls, and those of tongue, lips, and gingiva were collected from cancer subjects (histological grade; six well-differentiated, one moderate-differentiated, two poorly differentiated). Healthy and cancerous tissue samples were stored as formalin-fixed paraffin-embedded (FFPE) tissues [15, 16]. All FFPE samples were sliced into five mirror-image 3 μm thick sections. One section was stained with hematoxylin and eosin stain and visualized under a microscope, and the four remaining sections were labeled with a permanent marker and microdissected to isolate healthy and cancerous cells. The histological grade of cancer was identified by a pathologist (NK).

### Collection of oral epithelial cells

Samples recruited from Department of Otolaryngology, Faculty of Medicine, Chulalongkorn University, Bangkok, Thailand. There are 2 cohorts as followed:

Cohort 1. respresented as a discovery set, derived from patients during 2015–2016. This composed of oral rinse from 33 healthy controls and oral rinse as well as oral swab from 23 oral cancer subjects.

Cohort 2. represented as a validation set, derived from patients during 2016–2017. This composed of oral rinse from 54 healthy controls and oral rinse from 42 oral as well as 24 oropharyngeal cancer subjects.

Volunteers for this experiment were examined for their oral health. Subjects with no oral mucosal lesion, history of malignancy, or smoking behavior were included as healthy controls. All cancer subjects with oral or oropharyngeal cancer confirmed on the basis of incisional biopsy results of squamous cell carcinoma (SCC) were included in this experiment for comparisons. Oral mucosal cells were collected using oral rinse and oral swab techniques. Oral rinse was collected from oral and oropharyngeal SCC subjects and healthy controls, whereas oral swab was collected only from OSCC subjects. All demographic data, cancer histological grade and clinical stage were demonstrated in Additional file 2. For the oral rinse technique, the study subjects were required to gargle 10 mL of sterile 0.9% saline solution for 15 s. For collecting oral swab, a foam-tipped applicator (Puritan Medical Products, ME, USA) was applied to the OSCC lesion for 5–10 s. The oral rinse solutions and swab foams were stored in sterile tubes at 4 °C until needed for DNA extraction.

### DNA extraction and bisulfite conversion

DNA was extracted from healthy and cancerous cells using QIAamp DNA FFPE Tissue Kit (Qiagen, Valencia, CA, USA). The absorbance of extracted DNA was measured using NanoDrop (ND-1000 Spectrophotometer, NanoDrop Technologies, Wilmington, DE, USA) at 260 and 280 nm to verify its purity and concentration. Subsequently, DNA was subjected to bisulfite conversion using EZ DNA Methylation-Gold™ Kit (Zymo Research Corporation, Irvine, CA, USA) [17, 18] following the manufacturer’s instructions. Treatment of DNA with bisulfite converts unmethylated CG dinucleotide to TG but leaves the methylated CG dinucleotide (CpG sites) unaffected.

### Pyrosequencing and real-time PCR

Primers for polymerase chain reaction (PCR) amplification of DNAs and sequencing of PCR products were designed using Pyromark Assay Design software (Qiagen, Valencia, CA, USA). DNAs were amplified using the forward (5′-GGG GTT TTT AGA GTT GTA GAT TTT TGA-3′) and reverse (5′-CCA AAA ATA AAC TCC ACA AAA TAA ATC-3′) primers and sequenced using the sequencing primer (5′-TTT AGA GTT GTA GAT TTT TGA TTT G-3′). Pyrosequencing was then performed using the Pyromark Q96 ID platform (Qiagen, Valencia, CA, USA) to determine the methylation level according to the manufacturer’s instructions.

For screening purposes, real-time PCR was established, as it is easier than pyrosequencing and suitable for a large sample size. We designed the real-time methylation-specific PCR, which was performed using the Sensi-FastTM SYBR® Low-Rox Kit (Bioline, Alexandria, NSW, Australia). PCR reactions were prepared in a volume of 20 μL containing 10 μL of 2X Sensi-FastTM SYBR® Low-Rox reagent, 0.8 μL of 500 nmoles of each gene-specific forward and reverse primers and 1 μL of bisulfite-treated DNA template; the remaining volume was adjusted by adding milliQ DNase-free sterile water. Two gene-specific primer pairs were used in this experiment: one pair was specific to the target gene in its methylated state (met forward primer, 5′-ATT CGG GGA TTC GGG ATT C-3′ and met reverse primer, 5′-GAC GAC CCA TCT AAA AAA AAC TCG-3′) and the other pair was specific to the target gene in its unmethylated state (unmet forward primer, 5′-GGA TTT GGG GAT TTG GGA TTT-3′ and unmet reverse primer, 5′-CAC TCA AAC CAC CAC CTA ACA-3′). Real-time PCRs were performed in duplicates using an Applied Biosystem® 7500 Real-Time PCR System (Thermo Scientific™, Waltham, MA, USA). No template control and positive control were included in each PCR to observe for possible contamination. The PCR conditions were as follows: 45 cycles of denaturation at 95 °C for 2 min followed by annealing at 60 °C for 30 s. Fluorescence signals from the amplified product were detected at the end of the annealing step. A melting curve was generated to determine the specificity of the primers. The threshold cycle (C_t_) of the amplified methylation products was detected. Non-methylation-specific PCRs were used as an internal control to adjust C_t_ values. C_t_ values of all samples were used to calculate DNA concentrations on the basis of the standard curve prepared using 10-fold serial dilutions of universal methylated DNA standard (Zymo Research Corporation, Irvine, CA, USA) (10, 1, 0.1, 0.01, and 0.001 ng/μL).

### Statistical analysis

All statistical analyses were conducted using SPSS software for Windows version 22 (SPSS Inc., Chicago, IL, USA). Independent sample *t*-test was performed to determine differences among all groups except matched cases of oral rinse and swab samples, for which the paired *t*-test was used. *p*-values < 0.05 were considered statistically significant. A receiver operating characteristic (ROC) curve was generated to determine the diagnostic ability of gene methylation level to differentiate between epithelial cells from oral rinse and swab samples and those from healthy controls.

## Results

### Methylation microarray analysis

Following the bioinformatics approach (Fig. 1a), 27,578 CpG sites were identified from the GEO repository that showed differential methylation between healthy and cancerous cells. The average methylation percentage was plotted for each differentially methylated CpG site. From a collection of 27,578 graphs, one graph showing the highest difference in methylation levels between healthy and cancerous cells was selected. This graph represented the CpG site, cg01009664 in the thyrotropin-releasing hormone (TRH) gene sequence (Fig. 1c).

### Pyrosequencing of microdissected FFPE samples

**Fig. 2:**
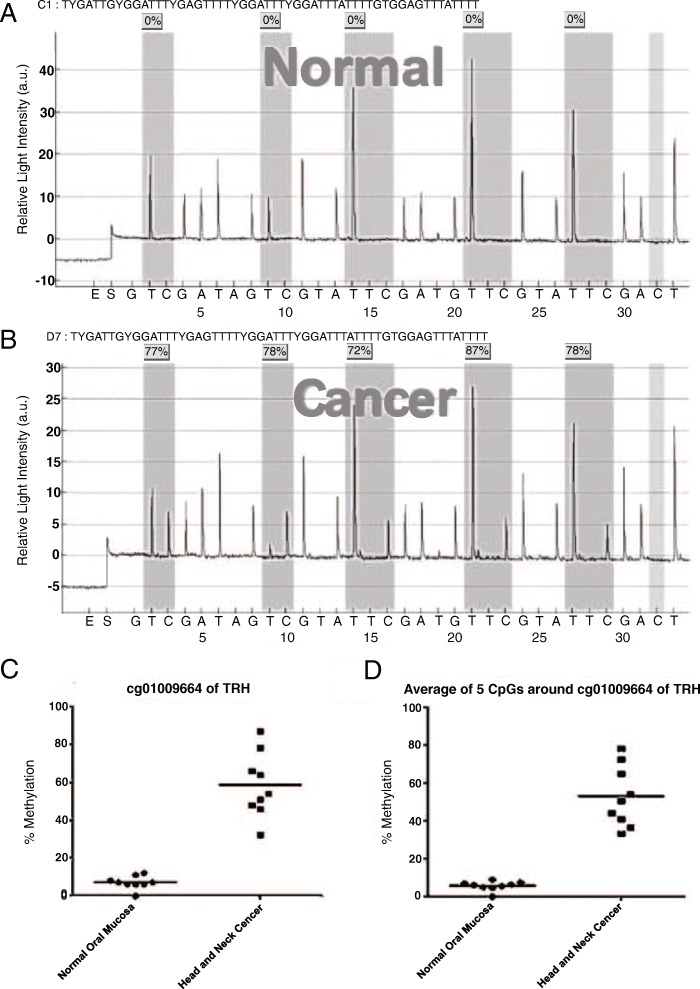
Pyrosequencing from microdissected tissue analyzed by unpaired t-test. **a** Example of pyrosequencing results in normal oral epithelia showed low level of methylation percentage in all 5 CpG surrounding cg01009664 of TRH. **b** Example of pyrosequencing results in OSCC showed high level of methylation percentage in all 5 CpG surrounding cg01009664 of TRH. **c** Percent methylation level cg01009664 of TRH (position 2) showed significantly higher in Head and Neck cancer comparing to normal oral mucosa. **d** Average percent methylation of 5 CpG surrounding cg01009664 of TRH showed significantly higher in Head and Neck cancer comparing to normal oral mucosa

DNA isolated from microdissected oral epithelial cells of nine healthy and nine OSCC FFPE samples were subjected to pyrosequencing for the validation of the CpG site, cg01009664. Pyrosequencing revealed five differentially methylated CpG sites surrounding cg01009664 in the TRH gene (Fig. 2a and b). The average methylation percentage of the nine samples at the second CpG site of TRH (cg01009664) in healthy cells (7% ± 1.14%) was significantly lower than that in cancerous cells (58.44% ± 5.68%) (*p* < 0.001; Fig. 2c). Similarly, the average of methylation percentage of nine samples at the five CpG sites surrounding cg01009664 in healthy cells (5.7% ± 0.85%) was also significantly lower than that in cancerous cells (52.96% ± 5.36%) (*p* < 0.001; Fig. 2d).

### Real-time PCR at cg01009664 of TRH in oral rinse and swab samples as a diagnostic test

**Fig. 3:**
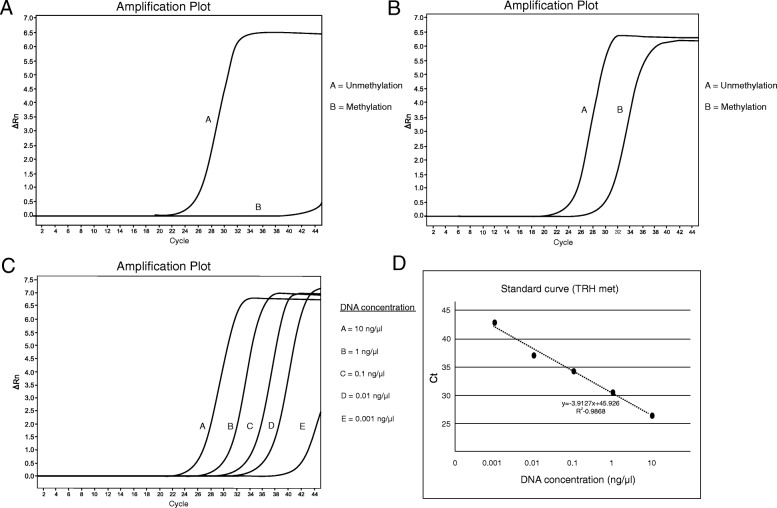
Real-time PCR amplification plot at cg01009664 of TRH in oral samples. **a** Example of graph from oral rinse sample of normal control showed only unmethylated product. **b** Example of graph from oral rinse sample of OSCC patient showed methylated and unmethylated product. **c** Ten-fold serial dilution for standard curve setting using universal methylated DNA (concentration 10, 1, 0.1, 0.01 and 0.001 ng/μL). **d** The standard curve of TRH methylation level for methylated DNA calculation. This method calculated methylated DNA under the formula; y = − 3.9127× + 45.926, y was cycle threshold (Ct) whereas x was methylated DNA concentration (ng/μL)

Next, we evaluated TRH methylation levels as a screening marker using real-time PCR. Oral rinse samples from healthy controls showed an amplification signal only for unmethylated TRH, whereas the amplification of methylated TRH was barely detectable (Fig. 3a). By contrast, oral rinse samples from OSCC subjects showed amplification of both methylated and unmethylated TRH (Fig. 3b). The standard curve was prepared using 10-fold serial dilutions (10–0.001 ng/μL) of control DNA samples (Fig. 3c) and used to calculate the concentration of methylated TRH samples based on their C_t_ values (Fig. 3d).

**Fig. 4:**
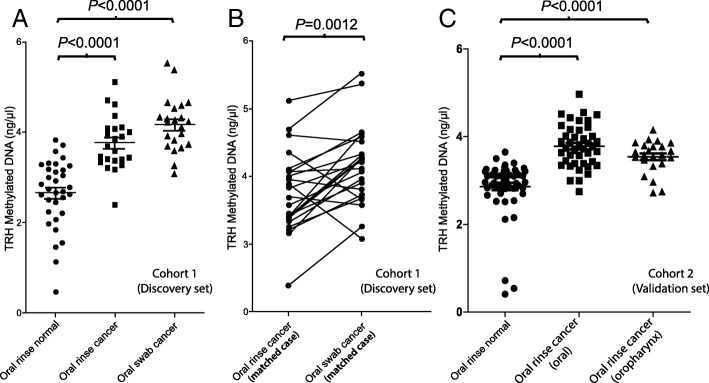
The amount of TRH methylated DNA (ng/μL) in oral rinse and oral swab samples. **a** Comparisons of methylated DNA from samples in cohort 1 (Discovery set). **b** Comparisons of methylated DNA between oral rinse and oral swab in individual cancer subjects (matched case) from cohort 1. **c** Comparisons of methylated DNA from samples in cohort 2 (Validation set)

In cohort 1, the average TRH methylation level of oral rinse samples collected from healthy controls was significantly lower than that of oral rinse samples collected from OSCC subjects (2.66 ± 0.76 ng/μL vs. 3.77 ± 0.60 ng/μL, respectively; *p* < 0.001) (Fig. 4a, Additional file 2). Additionally, the average TRH methylation levels of oral swabs were significantly higher than that of oral rinses in the same case significantly (4.17 ± 0.58 ng/μL vs. 3.77 ± 0.60 ng/μL, respectively; *p* = 0.0012) (Fig. 4b, Additional file 2).

In cohort 2, the average TRH methylation level of oral rinse samples collected from healthy controls was significantly lower than that of oral rinse samples collected from OSCC subjects same as cohort 1 (2.86 ± 0.64 ng/μL vs. 3.78 ± 0.48 ng/μL, respectively; *p* < 0.001) (Fig. 4c, Additional file 2). Moreover, the average TRH methylation levels of oral rinse samples collected from oropharyngeal SCC subjects was significantly higher than that of healthy controls same as OSCC subjects (3.54 ± 0.37 ng/μL vs. 2.86 ± 0.64 ng/μL, respectively; *p* < 0.001) (Fig. 4c, Additional file 2). The average TRH methylation levels of oral rinse samples collected from oropharyngeal SCC subjects was a little bit lower than OSCC subjects but not significantly different (3.54 ± 0.37 ng/μL vs. 3.78 ± 0.48 ng/μL, respectively) (Fig. 4c, Additional file 2).

All cancer samples, there were no significant differences in TRH methylation levels between different genders, ages, stages, and tumor grades (data not shown).

### Receive operating characteristics analysis (ROC analysis)

To evaluate the potential use of TRH methylation as a biomarker for detecting OSCC and oropharyngeal SCC, we performed ROC curve analysis. This method is an effective statistic for assessing the diagnostic test. It compromises the value of sensitivity and specificity, determining the best cut-off points, which receives the highest true positive rate along with lowest false positive rate. The area under the curve (AUC), cutoff value, sensitivity, and specificity for cohort 1, cohort 2 and cohort 1 + 2 can be seen in Table 1. Combination of oral rinses collected from OSCC subjects in cohort 1 and 2, ROC analyses using the cutoff TRH methylation value 3.31 ng/μl revealed a sensitivity of 86.15%, a specificity of 89.66% and an AUC of 0.93. At the same cutoff value oral rinse collected from oropharyngeal SCC, showed a sensitivity of 82.61%, a specificity of 92.59% and an AUC of 0.88. The ROC curves also indicated that oral swab samples (sensitivity of 91.30%, a specificity of 84.85% and an AUC of 0.97) were superior to oral rinse samples for detecting OSCC.

**Table 1:** Receive operating characteristics analysis when using cut-off ≥3.31 ng/μl

	Comparative data	Area Under the ROC Curve (AUC), Standard Error (95% CI)	Sensitivity% (95% CI)	Specificity% (95% CI)	Likelihood ratio
Cohort 1	Oral rinse from oral cancer subjects: Oral rinse from healthy controls	0.90, 0.043 (0.82 to 0.98)	82.61 (61.22 to 95.05)	84.85 (68.10 to 94.89)	5.45
	Oral swab from oral cancer subjects: Oral rinse from healthy controls	0.97, 0.02 (0.93 to 1.01)	91.30 (71.96 to 98.93)	84.85 (68.10 to 94.89)	6.03
Cohort 2	Oral rinse from oral cancer subjects: Oral rinse from healthy controls	0.93, 0.03 (0.88 to 0.99)	88.10 (74.37 to 96.02)	92.59 (82.11 to 97.94)	11.89
	Oral rinse from oropharyngeal cancer subjects: Oral rinse from healthy controls	0.88, 0.05 (0.77 to 0.98)	82.61 (61.22 to 95.05)	92.59 (82.11 to 97.94)	11.15
Cohort 1 + 2	Oral rinse from oral cancer subjects: Oral rinse from healthy controls	0.93, 0.02 (0.88 to 0.97)	86.15 (75.34 to 93.47)	89.66 (81.27 to 95.16)	8.33

*CI* confidence interval

## Discussion

Our bioinformatics approach revealed site-specific methylation at cg01009664 of the TRH gene, a novel marker for OSCC screening. TRH methylation has been reported in pancreatic cancer, lung cancer and clear cell renal cell carcinoma [19–21]. To the best of our knowledge, this is the first study to demonstrate TRH methylation in OSCC. Pyrosequencing of FFPE samples revealed five differentially methylated CpG sites in the promoter TRH gene. The second CpG site from the 5′ end of the TRH gene, cg01009664, showed the highest difference in methylation levels between healthy and cancerous cells. Moreover, the addition pyrosequencing experiment from DNA of WSU-HN17 cell line (head and neck cancer cell line, provided by Dr. J. Silvio Gutkind) also presented a very high methylation level at these sites in the same manner (Additional file 3). Not only the second CpG site but also the other surrounding CpG sites in the TRH gene showed similar methylation levels. These CpG methylations at promoter regions reportedly affect gene transcription and repress gene expression [22–25]. Although the function of TRH in cancer is currently unknown, methylation of the TRH gene in cancerous cells revealed in this study and previous studies [19–21] suggest that TRH functions involving carcinogenesis as a tumor suppressor gene [26]. Further researches need to validate the possibility, which may be applied as a cancer therapeutic target in the future.

TRH methylation showed high sensitivity and specificity for screening OSCC and oropharyngeal SCC. Using real-time PCR, TRH methylation was detected in cancer epithelial cells collected using both oral rinse and oral swab. However, oral swab technique showed higher sensitivity and specificity than oral rinse. This explains the collection of a significantly greater number of epithelial cells from cancer lesions using oral swab technique than using oral rinse. Only one case of false negative in oral swab may have arisen from technique that can be swab only covering necrotic tissue over the lesion. Despite the higher sensitivity of oral swab technique, oral rinse is more suitable for screening large numbers of subjects. Biopsy is an invasive technique; methylation status of TRH CpG sites demonstrates the potential to serve as an OSCC and oropharyngeal SCC noninvasive biomarker. For example, in cases where the occurrence of OSCC and oropharyngeal SCC is uncertain, it may be used as an additional test for confirmation, while using this technique in oral rinse samples may be suitable for OSCC screening for a larger population.

Comparing to cohort 1 and cohort 2, the average of TRH methylation level of oral rinse samples collected from healthy controls in cohort 1 and cohort 2 were very was nearly similarly level as well as oral rinsed from OSCC subjects in both cohort 1 and 2. That demonstrated the reliability of real-time PCR test and confirmed the consistency of this biomarker.

Unfortunately, this study did not find any difference between TRH methylation level and clinical stage or histological grade. In the future, we plan to validate the TRH biomarker in a larger sample size. Our objective is to categorize smoking epithelial cells and precancerous lesions according to their methylation percentages. This will assist in optimal treatment and prognosis of cancer patients. Currently, the determination of methylation percentage involves many laboratory procedures, including microdissection, bisulfite treatment of DNA, and PCR. In the future, we aim to develop a simple kit to facilitate the detection of OSCC from oral rinse, which will enable the detection of cancer cells at an early stage.

## Conclusion

Using a bioinformatics approach, we identified and validated TRH site-specific methylation as a candidate biomarker for OSCC detection.

## Additional files

Additional file 1: Microsoft Office Excel displayed average methylation percentage of each methylated CpG sites of each sample from the seven GSEs. (XLS 8189 kb)

Additional file 2: Demographic data and TRH methylation percentage in screening experiments. (XLS 29 kb)

Additional file 3: Pyrosequencing results of WSU-HN17 showed high level of methylation percentage in all 5 CpG surrounding cg01009664 of TRH. (EPS 1156 kb)

## Abbreviations

AUC: Area under curve
CpG: CG dinucleotide
FFPE: Formalin-fixed paraffin-embedded tissue
GEO: Gene expression omnibus
HNSCC: Head and neck squamous cell carcinoma
NCBI: National center for biotechnology information
OSCC: Oral squamous cell carcinoma
PCR: Polymerase chain reaction
ROC: Receiver operating characteristic
SCC: Squamous cell carcinoma
TRH: Thyrotropin-releasing hormone
